# Building a Realistic Virtual Luge Experience Using Photogrammetry

**DOI:** 10.3390/s25082568

**Published:** 2025-04-18

**Authors:** Bernhard Hollaus, Jonas Kreiner, Maximilian Gallinat, Meggy Hayotte, Denny Yu

**Affiliations:** 1 Department of Medical, Health & Sports Engineering, Management Center Innsbruck, Maximilianstraße 2, 6020 Innsbruck, Austria; jonas.kreiner@mci.edu (J.K.); maximilian.gallinat@mci.edu (M.G.); 2 Université Côte d’Azur, LAMHESS, Campus STAPS, 261 bd du Mercantour, 06205 Nice, France; meggy.hayotte@univ-cotedazur.fr; 3 School of Industrial Engineering, Purdue University, 315 N. Grant St, West Lafayette, IN 47907, USA; dennyyu@purdue.edu

**Keywords:** virtual reality, luge, skill training, photogrammetry, gamification, acceptance

## Abstract

Virtual reality (VR) continues to evolve, offering immersive experiences across various domains, especially in virtual training scenarios. The aim of this study is to present the development of a VR simulator and to examine its realism, usability, and acceptance by luge experts after an experiment with a VR simulation. We present a novel photogrammetry sensing to VR pipeline for the sport of luge designed with the goal to be as close to the real luge experience as possible, potentially enabling users to learn critical techniques safely prior to real-world trials. Key features of our application include realistic terrain created with photogrammetry and responsive sled dynamics. A consultation of experts from the Austrian Luge Federation led to several design improvements to the VR environment, especially based on user experience aspects such as lifelike feedback and interface responsiveness. Furthermore, user interaction was optimized to enable precise steering and maneuvering. Moreover, two learning modes were developed to accommodate user experience levels (novice and expert). The results indicated a good level of realism of the VR luge simulator. Participants reported scene, audience behavior, and sound realism scores that ranged from 3/5 to 4/5. Our findings indicated adequate usability (system usability score: 72.7, SD = 13.9). Moderate scores were observed for the acceptance of VRodel. In conclusion, our virtual luge application offers a promising avenue for exploring the potential of VR technology in delivering authentic outdoor recreation experiences that could increase safety in the sport of luge. By integrating advanced sensing, simulations, and interactive features, we aim to push the boundaries of realism in virtual lugeing and pave the way for future advancements in immersive entertainment and simulation applications.

## 1. Introduction

Sledding sports, including activities such as luge, tobogganing, skeleton, and bobsledding, likely began in the 19th century [1]. Since its start, lugeing has grown significantly as a sport among athletes, but it has also gained popularity and interest among the general population as a recreational activity—needing only a sled and hill to participate. Despite the public perception of lugeing as safe [2], the risk of injury in tobogganing is higher than in other winter sports like skiing. In the United States, emergency departments average 22,048 cases annually related to luge injuries [2]. The most common injuries in Austria in the years from 2008 to 2018 were head (21%) and leg/foot injuries (39%) [3]. Head injuries strongly correlated with the fact that the injured person did not wear a helmet [3]. Spine and head injuries can be particularly serious. In 2023, nine percent of tobogganing accidents led to head injuries, according to the Austrian Road Safety Board [4]. Injuries impact people of all ages, beginners and experts alike, with the highest prevalence among those at the intermediate technical level [3,4,5]. With increasing interest, erroneous safety perceptions, and high rates of injuries among the general population, there is a need to find the causes of the frequent accidents and counteract them.

The contributing factors and causes for injuries in lugeing are well documented. The majority of injuries are due to crashes, impacts against obstacles, and collisions [3,4]. A key danger is speed, where even recreational sledders can reach speeds of over 40 km/h [6]. Collisions at high speeds or falls followed by subsequent collisions with standing objects such as trees and ski lift poles (collisions with other persons are less common [1]) are commonly observed [5]. Other factors include lighting (e.g., lugeing at night), snow conditions, alcohol, and lack of personal protective equipment [5,7,8,9].

Given the common perceptions of safety and the adoption of the activity by the general population, the training of the technical skills and awareness of the injury risks can be useful strategies for improving the safety of the sport. Many may try the sport of luge with no formal training due to its perception of safety and accessibility. The current strategies for learning how to sled are knowledge-based (reviewing training material and websites) and/or a learn-by-doing approach (with or without a coach). However, knowledge-based training lacks the physical interactions that are critical and not often intuitive for steering and controlling the sled as it requires both complex feet and hand/rope coordination [10]. In contrast, learning in the real environment can expose novices to injuries as they learn while lugeing at potentially high speeds surrounded by trees, precipices, and barriers together with other users of the recreation space. Although this risk is minimized with the presence of coaches, trained and certified coaches are limited and costly and therefore not always accessible to the large number of people interested in the sport of luge [11]. There is a need for the ability to provide easy access to lugeing knowledge and experience in a safe environment to avoid the currently observed high rates of accidents.

Virtual reality (VR) offers an immersive platform that can be used in training across multiple domains [12]. There is supportive evidence that VR can improve psychomotor performance and knowledge acquisition [13]. With the reduced costs and proliferation of consumer-grade devices, the opportunities provided by this modality of training can also be made accessible to an increasing proportion of the population [14]. In fact, there have been multiple skill acquisition use cases for VR in the context of sports and other winter recreation activities [15,16,17]. However, the effectiveness of VR for training users to sled in the real world is limited to the validity of the simulation.

To the best of our knowledge, actual sledding simulators are not available in VR, resulting in low validity. Since the known contributors to injuries during lugeing are closely related to both technical skills (sled control) and the dynamic and highly variable environmental conditions, it is necessary to develop a realistic VR environment. We proposed a framework using state-of-the-art photogrammetry and virtualization concepts and methods to develop a realistic, acceptable, and user-centered VR training environment.

The aim of this paper is to present the proposed framework and to examine its realism, usability, and acceptance by luge experts after an experiment with the VR simulation.

To address the challenge of accessible realistic training for luge safety, the main contributions of this study are summarized as follows:

- A novel photogrammetry sensing to VR pipeline for the sport of luge.
- VR parameters (visual and physics modeling) needed to achieve realism in the context of safety and training.
- User studies with subject matter experts (members of the Austrian luge team).
- The first VR simulator for luge designed for realism, acceptance, and usability needed for training.

## 2. Material and Methods

The methods are presented in two sections, the VR game development part and the beta testing and expert consultation part.

### 2.1. VR Game Development

Since a game can only be developed if you know the requirements, the authors decided to conduct an acceptability study before the game development started. This acceptability study led to a proof of concept of VRodel, followed by several versions of the VRodel game.

#### 2.1.1. Acceptability of the Proof of Concept

Several steps were followed to develop the VR game. First, an acceptability study was carried out [18]. In this way, it is possible to minimize the risk of developing a game that does not address the needs and requirements of the users and stakeholders. The results demonstrated good acceptability of the proof of concept for the VR game for safety education (i.e., presentation text with a figure). One of the major outcomes concerned the priority features to include, which were realistic visual details, and realistic interactions in the virtual environment. Consequently, particular attention was paid to developing a simulation perceived as being as real as possible. This result aligned with the findings of [19,20]. To address this requirement, we implemented techniques novel to VR luge training, including the use of photogrammetry and physics modeling.

#### 2.1.2. Photogrammmetry

Creating a virtual world from the ground up that is perceived as real is possible, but not efficient as the developed environment may potentially miss elements from the real environment. Mapping a real luge track into a virtual copy of it is more effective, especially in Tirol, Austria. According to the Austrian Luge Federation, Tirol has more than 200 luge tracks [21]. Making a virtual copy of one of them can be performed by the means of photogrammmetry with post-processing of the virtual model. Based on this boundary condition, the luge track of Kühtai in the Stubai valley was chosen [22]. However, the proposed method can be scaled to other tracks as needed.

For the given track, it was necessary to record the image and video material to perform photogrammmetry. On the 22 March 2023, the recordings were gathered during dusk. The equipment used is shown in Table 1.

**Figure 1:**
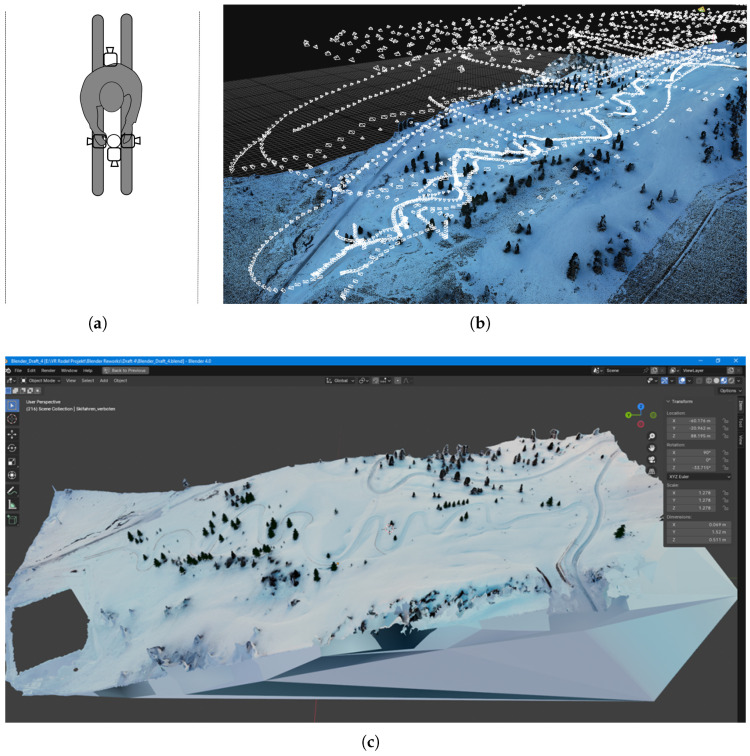
The experimental setup for the data recording with the four Hero 11 cameras (**a**) and the flight plan of the two drones (**b**). Based on the drone’s data, a first model was created in the software Reality Capture with a photogrammetry approach. This model was the further processed in Blender and is shown here (**c**).

The Phantom 4 was controlled via the flight planning tool DJI Terra. The accuracy of the drone’s position measurement was further improved by the utilization of a ground station, as listed in Table 1. As the snow was marked with a pink markerspray, alignment between videos and the geographical landmark of the ground station became possible. The video taken with the Phantom 4 was processed with MATLAB R2022b into images. The Mavic 3 gathered a series of images that did not need any further processing. In total, 2356 images were chosen from the Mavis 3 and Phantom 4 data for the photogrammmetry step. To obtain better details in the model close to the track, four cameras of the type GoPro Hero 11 were used. A skier equipped with the cameras descended the track, as illustrated in Figure 1. To obtain images, the four videos were processed with the same MATLAB code as the drone videos. Since the contribution of these images did not refine the mesh of the model but made it worse, they were not considered for the creation of the model. In Figure 1, the first model is shown. It was created with the Reality Capture software.

The model of the track was not sufficient to create a virtual world that met our goal of realism. Therefore, this model was embedded into another model to capture the far field. The far field model covered more area of the Stubai valley, with the mountains around the track, side valleys, smaller hills, and rivers. Photogrammetry was not the proper method to transfer this area into a virtual world as the amount of data to be processed exceeds the possibilities that were available. Therefore, another approach was chosen. Based on a height map of the area from a geo−information service, the terrain could be created with the Unity Terrain Tools. The details of the far field model were less accurate in comparison to the photogrammetry model, but as they will not appear close to the avatar within the simulation, the accuracy was sufficient. The two combined models were the foundation for the resulting virtual world.

#### 2.1.3. Creating the Virtual World

**Figure 2:**
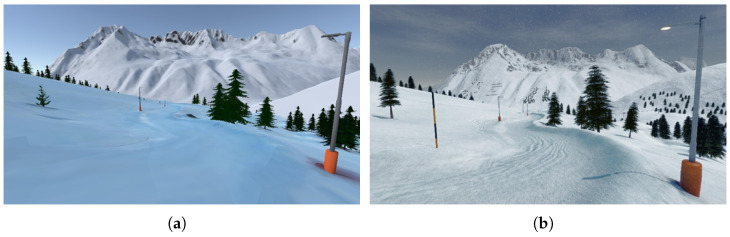
Starting from a nearly unenhanced photogrammetry model (**a**), an enhanced virtual world was created (**b**).

Within Blender, the two models were combined into a virtual world. The combined model did not have the required quality with respect to realism. Therefore, further steps were necessary to improve the model to an acceptable level. In the first step, the far field needed a corresponding texture. The texture was created by using shaders. We also included assets from the Unity asset store and custom-made assets. The ropeways within the valley and the forest and snow on the mountains had to be created and included. The software packages that were used to create the corresponding shapes, textures, images, items, etc., were GIMP, Adobe Substance Painter, and Blender. The track model also needed visual improvements. The nature of the photogrammetry approach is based on a static scene. Trees, snow, and any other objects that were not static usually appear bulky within the models. As these objects do not appear as real as they have to, these objects were replaced and optimized in their appearance. Figure 2 shows the improvements made.

After improving the models for visual realism, we focused on the second aspect that affects the perceived realism, which was the steering of the sled.

#### 2.1.4. Physics in the Game

To be able to steer a sled in the virtual world, it was necessary to create a physical model, especially for the sled–snow interaction. There are two main aspects in the physical modeling that were considered so that the steering was perceived as realistic. The first was tribology and air drag. The second was the weight distribution on the sled in combination with the pulling forces on the rods.

Unity uses a Coulomb friction model by default that is defined in the PhysiX physics engine of NVIDIA. The model is based on a static friction coefficient and a dynamic one. Choosing the right values for the friction coefficients is key for a virtual experience close to reality. In general, the friction coefficients range from less than 0.01 to more than 0.15 and highly depend on various conditions like snow humidity, snow temperature, steel surface temperature, relative velocity, snow texture, and various other parameters. Modeling all these conditions in the virtual world results in a highly complex model. Whether a more complex friction model directly affects the perceived realism is unknown. Therefore, a simple modeling approach for the friction coefficients was chosen, with the option to increase the model complexity if required by the end user. By combining the findings of [23,24,25,26,27], only a dynamic friction coefficient was used for the virtual world, as the static coefficient is too low to have a meaningful impact. The model uses $F_{f}=\mu \cdot m\cdot g\cdot cos\left(\phi \right)$ with $F_{f}$ as force due to friction, μ as the friction coefficient, *m* as the mass (given with 70 kg), *g* as the gravity constant, and ϕ as the current angle of the track. Depending on the surface the sled is currently on, the friction coefficient is adapted according to Table 2.

Air drag also contributes as a braking force along with $F_{f}$. In an ideal simulation, it would be necessary to consider various inputs like wind, body, and luge posture orientation and dimensions. The level of model complexity has to be as high as needed for good perceived realism. A general air drag force model can be stated with $F_{a}=c_{d}\cdot A_{a}\cdot v_{s}^{2}$, with ($F_{a}$) being the air drag force, $c_{d}$ as the drag coefficient, and $A_{a}$ as a constant effective area relevant for air drag. $A_{a}$ was estimated with 0.53 m^2^. $c_{d}$ was estimated by considering drag coefficients in skeleton, skiing, and ice track luge [28] with 0.8. Assuming a speed condition of 8 m s^−1^, like [26], the air drag force can be estimated with approximately 26.6 N. The combination of air drag and Coulomb friction directly affects the maximum speed for a given slope angle. Especially for low speeds, air drag does not have a significant contribution to the total braking force. In a curve, the braking forces are different from a straightaway. For simplicity in the modeling, additional curve friction was not considered.

Riding through a curve has to be a consequence of steering. Steering a real luge can be performed by distributing the athletes’ weight on the luge and pulling on the rods. This would offset the steel runners and steer the luge. For the game, two inputs were considered: the controller and headset positions. Depending on the players’ skill level, they can choose either the standard or the professional steering mode. The standard mode uses only the distance between the left and right controller origins as a weighted input for steering torque. This means that only the pulling motion impacts the offset in the steel runners in a simplified way, but no weight distribution is considered. The steering torque rotates the avatar and luge on the slope according to the input.

Other actions can also be performed to steer the sled. A pedaling motion with both controllers creates an additional force in the current movement direction of the luge. This is a typical motion an athlete would perform to start from a standstill position. The higher the pedaling speed, the more force is created. Braking is initiated by pulling both controllers toward the head. This motion reflects the real motion that an athlete would perform when braking the luge. Below a distance threshold of 30 cm, braking is activated with a constant braking force until the avatar decreases the speed to 0 m s^−1^. Alternatively, the player can use the index finger buttons of the controller to emulate a break with their left or right foot. By pushing the buttons, a constant torque and force is introduced to the luge that breaks the luge and simultaneously rotates it. If both buttons on both controllers are pushed at the same time, the torque becomes zero, and only the braking force remains. This approach also reflects how real braking with the feet would unfold while sledding.

The professional steering mode is more complex. The natural steering motion of elite athletes in natural luge can be seen as a process that follows the following steps:

1. Grab the diagonal rod. In this phase, the athlete would grab the rod that is attached to the left steel of the luge with their right hand or vice versa.
2. Pull the grabbed rod. In this phase, the rod is pulled. The pulling is indicated by pushing the middle finger button. This position of the controller is then set as the reference position. The further the controller is pulled away from this reference position, the more the pulling motion contributes to the torque that acts on the luge.
3. Leaning into the curve. The IMU in the headset measures how far the player leans. Leaning into the curve can be interpreted as weight distribution change. The leaning angle directly contributes to the torque that acts on the luge in a linear fashion. The more the player leans into the curve, the stronger the torque will be.

As can be seen in the process above, the torque that rotates the luge and the avatar comprises the pulling motion and the weight distribution. The pulling motion is weighted with a share of 50%; the weight distribution is weighted with 50% as well, so steering with leaning is possible without pulling and vice versa. This also reflects the typical movements during a luge ride on a natural track.

#### 2.1.5. Game Modes

The virtual world was embedded in a VR application developed in Unity. The physics engine of Unity enabled the development of the sled steering. As potential users range from young novice athletes to experts, with extensive experience and several years of professional tobogganing, the application was split into two modes of games (standard and expert).

In the process of adjusting and optimizing routines between the developers and experts from the Austrian Luge Federation, the various ways to steer a sled were discussed and analyzed. As mentioned, the range of potential users is very wide and has specific requirements. Therefore, the major difference between the two game modes was sled control to adapt to the skills of advanced and novice users.

To implement this virtual world, we utilized the Meta Quest 2 VR goggles and the corresponding left- and right-hand-held controllers connected with a computer via a link cable. As the final virtual world needed to be rendered in a way to enable a smooth game experience, the computer was equipped with an NVIDIA RTX4070 graphics card. Based on this setup, the design of the game mode was encountered.

The standard mode considers the following inputs for controlling the sled:

- Index finger buttons (left and right) for braking/steering with the feet.
- Position in space of the left and right controller.
- The Y button respawns the avatar on the track.
- The X button resets the camera view.

The steering was also introduced with a tutorial video for all testers that can be viewed here: https://youtu.be/XzoTN2ieDQo accessed on 13 April 2025. As soon as the first virtual world was combined with the game mechanics, a virtual simulation called VRodel was created and ready for testing.

### 2.2. Beta Testing and Expert Consultation

As the given goals were to assess VRodel for realism, usability, and acceptance, it was reasonable to host testing events to collect the data. Three events were used to collect the data. In the first one, two experts from the Austrian Luge Federation gave very detailed feedback for the developers to optimize the simulation in expert interviews. The two experts were recruited from the Austrian Luge Federation. Both had more than 15 years of professional luge experience at that time. They have participated in several world cup seasons and Olympic winter games, winning some of the races. In the second, we hosted an exhibition stand at the Alpine Fair 2023 in Innsbruck together with the Austrian Luge Federation. The goal was to obtain a first impression of the beta testers that can be used by the developers for further improvements. The recruitment of the participants was performed actively. Visitors of the exhibition stand were asked if they wanted to test VRodel. Interested people could test VRodel after an introduction to the system and signing an informed consent form. The level of experience in luge ranged from novice users to experts. The testers were asked for feedback and their opinion for improvement after they experienced VRodel. The results obtained from these two events were not collected systematically and were only used directly to improve the quality of the game.

The last and final event was hosted as a competition within the team of the Austrian Luge Federation. Anybody from the team was offered to participate in the competition. In total, 15 people wanted to participate in the competition. All of them were either part of the professional athletes of the luge world cup team or the coaching staff. The experience of them was not queried individually, as the fact that they are professional athletes or part of the coaching staff of a world cup team implies that they are experts in the field of luge. Within the competition, all participants were instructed to the steering mechanisms of the expert mode. All participants had one training run to get used to the steering and a second run that counted for the competition. After the two runs, the participants filled out a survey with questionnaires about the realism, usability, and acceptance of VRodel. This study was ethically approved (number: [blind for review]). All participants gave their electronic consent before participation. The German VR Simulation Realism Scale [29] was adapted to the VRodel context to measure scene realism (5 items; α = 0.86), audience behavior realism (4 items; α = 0.86), and virtual sound realism (1 item) on a 5-point Likert scale from 1 (strongly disagree) to 5 (strongly agree). Cronbach’s alphas indicated good internal consistency [30]. The 10-item of the German System Usability Scale (SUS) [31] was used to calculate the SUS score based on a combination of the ratings using an algorithm. The acceptance of VRodel was measured with the same adapted questionnaire as the previous acceptability study [18] based on the German version of the Unified Theory of Acceptance and Use of Technology-2 [32,33]. Performance expectancy (4 items; α = 0.92), effort expectancy (4 items; α = 0.68), social influence (3 items; α = 0.81), facilitating conditions (4 items; α = 0.65), hedonic motivation (3 items; α = 0.78), price sensitivity (5 items; α = 0.65), habit (4 items; α = 0.66), and behavioral intention (3 items; α = 0.71) were measured on a 7-point Likert scale from 1 (strongly disagree) to 7 (strongly agree). Cronbach’s alphas indicated adequate internal consistency [30]. Demographics were also collected, including age, sex, frequency per year of sledding, expertise, and their use of VR. Descriptive statistics were computed to examine the realism, usability, and acceptance of VRodel. In addition, Spearman’s correlations were computed to examine the relationships between the realism, usability, and acceptance of VRodel. According to the literature [34], a correlation of less than 0.1 is considered trivial, a correlation between 0.1 and 0.3 is small, a correlation between 0.3 and 0.5 is moderate, a correlation between 0.5 and 0.7 is large, and a correlation greater than 0.7 is very large to almost perfect.

## 3. Results

The results are presented in two parts. The first one focuses on the technical results and the second part focuses on the survey results.

### 3.1. VRodel Simulator

VRodel can be presented as a result of this paper and is summarized as a video under https://youtu.be/S1eEsOKdyoc accessed on 13 April 2025. It represents a virtual experience in the sport of luge. The scenario in this experience starts in a mountain hut, and the user has to go through a series of decisions before the luge simulation can be played. The first decision the user has to make is about whether safety instructions with a follow-up quiz on luge safety should be read and taken or if the game should be played right away. If the user chooses to play right away, the decision of whether to play or skip the tutorial has to be made. The safety instruction with the follow-up quiz is an introduction to signs that might occur on the luge track followed by a quiz about the signs. Independent of the previous decisions, the user has to pack a rucksack with the right gear for lugeing after the tutorial part. All of the starting tasks already address the overall goal to decrease the injury rate in sledding. The series of decisions ends with the last decision on which game mode the user wants to play in.

After the decisions, an avatar spawned directly at the start of the luge track. After a short countdown, the descent in the virtual world started. In most cases, the user steered the avatar down the luge track over the finish line within two to three minutes. Depending on the time, the user was listed on the leaderboard of the game.

### 3.2. Expert Consultation

This final version of VRodel was tested by a panel of 15 luge experts composed of 12 males and 3 females with a mean age of 34.9 (SD = 10.5) years. They recreationally sled an average of 4.3 (SD = 3.7) times a year. They ranked their luge expertise at 4.3/5 (SD = 1.4) and their frequency of VR usage at 3.0/5 (SD = 1.4). The data are listed in Table 3.

Scene realism received a mean score of 4.0/5 (SD = 0.6), audience behavior realism received a mean score of 3.7/5 (SD = 0.7), and virtual sound realism received a mean score of 3.0/5 (SD = 1.0). These results indicate acceptable levels of realism, with the sound realism scoring the lowest among the three constructs of realism. The SUS scores ranged between 50 and 97.5, with a mean score of 72.7 (SD = 13.9) indicating adequate usability.

Moderate scores were obtained for acceptance of VRodel. Performance expectancy, which refers to “the degree to which using a technology will provide benefits to consumers in performing certain activities” [35], was scored at a mean of 3.8/7 (SD = 1.4). Effort expectancy, which refers to “the degree of ease associated with consumers’ use of technology” [35], was scored at a mean of 5.3/7 (SD = 0.9). Social influence, which refers to “the extent to which consumers perceive that important others (eg, family and friends) believe they should use a particular technology” [35] obtained a mean score of 4.1/7 (SD = 1.0). Facilitating conditions, which refer to “consumers’ perceptions of the resources and support available to perform a behavior” [35], were scored at a mean of 4.7/7 (SD = 1.2). Hedonic motivation, which refers to “the fun or pleasure derived from using a technology” [35], was rated at a mean of 5.5/7 (SD = 1.0). Price sensitivity, which refers to “consumers’ willingness to pay for a specific product or service” [33], was rated at a mean of 3.1/7 (SD = 1.1). Habit, which refers to “the extent to which an individual believes the behavior to be automatic”, was scored at a mean of 3.0/7 (SD = 0.9). The behavioral intention to use VRodel was scored at a mean of 4.5/7 (SD = 1.0).

The correlation matrix is presented in Table 4. Significant large positive correlations were shown between system usability and virtual scene realism (r = 0.54, *p* < 0.05), audience behavior realism (r = 0.59, *p* < 0.05), and virtual sound realism (r = 0.52, *p* < 0.05). A very large positive correlation was shown between system usability and hedonic motivation (r = 0.77, *p* < 0.05). Hedonic motivation was also positively correlated with virtual scene realism (r = 0.59, *p* < 0.05) and audience behavior realism (r = 0.64, *p* < 0.05). No correlation was demonstrated between the other constructs of acceptance of VRodel and realism or system usability.

## 4. Discussion

Although our objective for realism was supported by the user study, the resulting VR development required some simplification of the physics modeling; however, these selected simplifications were reasonable for the given simulation of VRodel.

The tribology of a sled is a highly complex topic. In recent decades, research has focused on modeling the interaction of surfaces such as ice and snow with various types of steel [23,24,25]. The friction coefficient as the central parameter depends on the relative speed, the temperature of the surface and steel, the roughness of the surface, and many more conditions. Considering all the dependencies for the model would result in a nonlinear model of the friction coefficient. At the same time, various conditions can appear dynamically during sledding. Snow conditions may change drastically on a natural track from top to bottom. Some parts may be covered in ice and some parts have soft and wet snow, which results in a drastic change in the friction coefficient depending on the surface condition.

According to [25], friction coefficients on ice vary slightly within a typical temperature range of −25 ^∘^ to 0 ^∘^. The dependency on the relative velocity of ice and steel is given in a negative way, meaning higher velocities result in a lower friction coefficient. Ref. [25] also shows that cold and snow results in friction coefficients of approximately 0.15. Ref. [26] obtained friction coefficients below 0.007 for speeds of approximately 8 m s^−1^.

Experiments that lead to the friction coefficient for soft snow conditions have never been performed for sledding on natural tracks. In the sport of skiing, several experiments have already been carried out, e.g., by [27]. Their findings show friction coefficients on winter and spring snow in dry, frozen, and wet conditions with various speeds. The results indicate that frozen and dry conditions generally lead to lower friction coefficients and wet snow increases the friction coefficient independent of spring or winter snow. The findings can be partly adapted for sledding.

Based on the literature, there would have been more complex models to choose from in contrast to the simple model that was chosen for VRodel. For the game experience, the most important question is how steering is perceived. As steering was perceived to be acceptable in terms of realism, the simple model was preferred to more complex ones, although adding complexity to the model in the future is possible.

The creation of a virtual experience for lugeing based on images and photogrammetry in combination with game physics that reflect reality should have the potential to increase safety in the sport of luge. At the same time, there are limitations in the results, methods, and system in general that need further discussion.

The initial questions of this project were about realism, usability, and perceived acceptance. The VRodel game based on a photogrammetry approach needed high realism, which was a required feature based on previous work [18,36]. Realism is important, leading to more positive user responses and to a greater sense of presence [20,36]. More importantly, it can help the potential transfer of skills learned in VRodel to real sledding tracks.

The original goal to have a simulation that is perceived as real was met, as the three realism metrics underline (scene realism: 4.0/5, SD = 0.6; audience behavior realism: 3.7/5, SD = 0.7; and virtual sound realism: 3.0/5, SD = 1.0). The virtual sound realism is arguably the worst of the three metrics. This could be expected because the focus of the photogrammmetry approach is on the shape of the environment, not the sound. Therefore, the sound was created synthetically within the virtual world and is not based on real sounds of the environment around a luge track. At the same time, sound realism probably has a minor effect on steering skill training in VR. Therefore, it is acceptable to have the lowest realism score in the sound metric with respect to the overall goal.

The other two realism metrics should be further discussed in terms of the potential for optimization. One aspect that could be improved is the steering. Currently, the two hand-held controllers, which are common for the Meta Quest 2 VR goggles, are used as the main controllers. Refs. [37,38] focused on designing specific controllers that enhance the haptic interaction for given requirements. The same approach with a focus on the sport of luge could increase immersiveness and realism. The real mechanics in steering a luge comprises pulling motions with the arms and hands, pushing motions with the feet, and changing the center of gravity with respect to the luge. This can only be monitored partly with the existing controller setup. Including force places in combination with an instrumented luge would increase the realism on the steering side of the simulation. Therefore, we argue that it would also affect the realism metric and the audience behavior realism metric.

Within the virtual simulator world, there are many sport simulations with a higher degree of model accuracy (e.g., Assetto Corsa in the sport of car racing). Nevertheless, for the sport of luge, no virtual simulation comes close to the realism introduced with VRodel. Alternative simulations of this sport might be the game VR Luge, which is available on the Playstation VR World; Sleigh Champion Winter sports, which is available on the Google Play application store; and Totally Realistic Sledding VR, which is available on the Steam store. All of these games focus on the gaming experience and entertainment but do not emphasize realism and safety. They try to engage users by exploiting action effects, which are not realistic. VRodel is a game that focuses on realism to enable the learning of skills that are transferable to real lugeing. Therefore, it can be said that VRodel is the first simulation of the luge sport.

Reflecting on the method of photogrammetry, it can be argued that other methods could be used to recreate the virtual luge track. Several other VR game developers have exploited artificial intelligence to create virtual environments. The methods are very promising in this area, but as the goal was to copy a real luge track, this option was not considered. This project used a photogrammetry approach, although light detection and ranging systems (LIDARs) might have led to a more accurate model shape. Photogrammetry was chosen because it has low expenses, high availability, and is easier to post-process the initial model into a virtual world. This made the photogrammetry approach reasonable for the authors. To have sufficient accuracy in the shape of the model in comparison to the real shape, a ground station was included in the data acquisition. In this way, the photogrammetry method does not rely on features in the recorded images only but can rely on accurate position information of the drone as well. This can be supported as the datasheet of the RS2+ shows a horizontal accuracy of 7 mm + 1 ppm V and 14 mm + 1 ppm V vertically [39]. In addition, the usability of VRodel was proven to be adequate with a mean score of 72.7. This score is comparable to other VR simulations for physical activity [40].

The actual version of VRodel obtained moderate-to-low acceptance scores. Especially, price sensitivity and habit were not as good as the other metrics. This result could be concerning, but we also know from previous research that in Unified Theory of Acceptance and Use of Technology-2 (UTAUT2), not all the constructs are predictors of behavioral intention to use the technology [35,41]. Although price sensitivity and habit were significant predictors of behavioral intention in previous similar work [18], one can question their contribution to behavioral intentions in this study. Future studies would be needed to highlight the contribution of these variables to explaining behavioral intention to use VRodel on a larger sample.

The overall goal of the VR game should be to prevent injuries in sledding. At this stage, it cannot be said if it does, and it is up to future work to provide data on this open question of whether training with VRodel prevents injuries.

## 5. Conclusions

The results show that VRodel is perceived as a virtual simulation with high realism overall. In comparison to the state of the art in the sport of luge, it can be said that VRodel is the first luge simulation ever that is perceived close to reality. Some aspects of the simulation still have the potential to improve and therefore also potentially improve the perceived realism. Future work would design, develop, and implement an optimal controller for the simulation. The perceived realism and acceptance of the device would be improved by these improvements but should be examined in a future study.

## Figures and Tables

**Table 1:** Equipment.

Equipment	Identification	Configuration	Used for Virtual Model
Drone	DJI Mavic 3	Manual Flight	Yes
Drone	DJI Phantom 4	Automated Flight	Yes
Ground station	Reach RS2+	RTK	Yes
Cameras	4 · GoPro Hero 11	Ultra Wide Mode	No
Markerspray	-	Color: pink	Yes

**Table 2:** Friction coefficients depending on the track surface.

Surface	Friction Coefficient μ
Ice	0.01
Dry snow	0.05
Wet snow	0.12

**Table 3:** Mean scores of the perceptions of realism, usability, and acceptance of VRodel by each of the luge experts (n = 15), ^1^ measured using the German VR Simulation Realism Scale [29] on a 5-point Likert scale, ^2^ measured using the German System Usability Scale (SUS) [31] on a 5-point Likert scale, ^3^ measured using the German version of the Unified Theory of Acceptance and Use of Technology-2 [32] on a 7-point Likert scale, and ^4^ measured using the German extended version of the Unified Theory of Acceptance and Use of Technology-2 including the price sensitivity [33].

ID	Virtual Scene Realism ^1^	Audience Behavior Realism ^1^	Virtual Sound Realism ^1^	System Usability Scale ^2^	Performance Expectancy ^3^	Effort Expectancy ^3^	Social Influence ^3^	Facilitating Conditions ^3^	Hedonic Motivation ^3^	Price Sensitivity ^4^	Habit ^3^	Behavioral Intention ^3^
1	4.2	5.0	4.0	80.0	5.5	6.0	6.0	5.0	6.3	5.8	4.3	6.0
2	3.8	3.0	1.0	67.5	4.5	5.3	4.7	5.5	6.0	3.4	2.8	5.0
3	5.0	4.5	3.0	95.0	1.8	3.5	3.3	4.3	7.0	3.0	1.8	4.3
4	4.0	4.0	3.0	65.0	2.0	4.3	5.0	4.0	3.3	2.2	2.0	2.0
5	3.2	2.5	3.0	57.5	4.8	4.8	5.0	4.8	5.3	4.4	4.5	5.3
6	3.6	3.0	4.0	80.0	4.0	4.8	5.0	3.3	5.0	3.0	2.8	4.3
7	4.4	4.4	2.0	85.0	3.8	6.3	4.0	6.8	6.7	1.4	2.8	4.7
8	4.0	4.0	4.0	97.5	4.5	6.8	4.7	7.0	6.3	3.2	3.8	5.0
9	4.2	4.0	5.0	80.0	3.5	5.8	3.7	4.3	5.7	2.0	2.3	3.7
10	2.4	3.5	2.0	55.0	2.5	4.8	2.7	3.3	4.3	2.6	2.5	4.0
11	4.2	4.0	3.0	72.5	2.8	6.3	3.0	5.5	5.0	3.0	3.5	4.0
12	4.2	4.3	3.0	75.0	6.5	5.5	5.0	4.8	6.3	4.2	4.0	6.0
13	4.2	4.3	3.0	67.5	1.5	6.3	3.3	5.5	6.0	2.0	2.5	4.7
14	3.6	3.3	2.0	50.0	3.8	5.0	2.3	4.5	4.0	2.8	2.0	3.3
15	4.4	2.5	3.0	62.5	5.0	4.5	4.0	2.8	5.0	3.2	3.3	5.0
Average	4.0	3.8	3.0	72.7	3.8	5.3	4.1	4.8	5.5	3.1	3.0	4.5
Standard deviation	0.6	0.8	1.0	13.9	1.4	0.9	1.1	1.2	1.1	1.1	0.9	1.0

**Table 4:** Correlation matrix of Spearman’s rho for the metrics from Table 3, ^1^ measured using the German VR Simulation Realism Scale [29] on a 5-point Likert scale, ^2^ measured using the German System Usability Scale (SUS) [31] on a 5-point Likert scale, ^3^ measured using the German version of the Unified Theory of Acceptance and Use of Technology-2 [32] on a 7-point Likert scale, and ^4^ measured using the German extended version of the Unified Theory of Acceptance and Use of Technology-2 including the price sensitivity [33], * level of significance below 0.05, ** level of significance below 0.01.

	1. Virtual Scene Realism ^1^	2. Audience Behavior Realism ^1^	3. Virtual Sound Realism ^1^	4. System Usability Scale ^2^	5. Performance Expectancy ^3^	6. Effort Expectancy ^3^	7. Social Influence ^3^	8. Facilitating Conditions ^3^	9. Hedonic Motivation ^3^	10. Price Sensitivity ^4^	11. Habit ^3^	12. Behavioral Intention ^3^
1	-											
2	0.588 *	-										
3	0.174	0.212	-									
4	0.539 *	0.594 *	0.520 *	-								
5	−0.063	−0.234	0.156	0.037	-							
6	0.141	0.370	0.168	0.396	0.110	-						
7	−0.056	0.035	0.415	0.269	0.618 *	−0.055	-					
8	0.151	0.387	−0.097	0.404	0.086	0.831 **	0.045	-				
9	0.588 *	0.635 *	0.151	0.769 **	0.199	0.390	0.220	0.562 *	-			
10	−0.141	−0.167	0.156	−0.006	0.776 **	−0.092	0.562 *	0.071	0.200	-		
11	−0.075	−0.103	0.237	0.100	0.783 **	0.396	0.567 *	0.371	0.251	0.723 **	-	
12	0.162	0.097	0.111	0.227	0.759 **	0.246	0.583 *	0.360	0.599 *	0.748 **	0.808 **	-

## Data Availability

The data presented in this study are available on request from the corresponding author.

## Abbreviations

The following abbreviations are used in this manuscript:

etc.	et cetera.
e.g.	exempli gratia.
UTAUT2	Unified Theory of Acceptance and Use of Technology-2.
VR	Virtual reality.
